# Optimization of Lipase-Mediated Synthesis of 1-Nonene Oxide Using Phenylacetic Acid and Hydrogen Peroxide

**DOI:** 10.3390/ijms131013140

**Published:** 2012-10-12

**Authors:** Emilia Abdulmalek, Mahashanon Arumugam, Mahiran Basri, Mohd Basyaruddin Abdul Rahman

**Affiliations:** 1 Department of Chemistry, Faculty of Science, Universiti Putra Malaysia, 43400 UPM Serdang, Selangor, Malaysia; E-Mails: shanons1986@yahoo.com (M.A.); mahiran@science.upm.edu.my (M.B.); basya@science.upm.edu.my (M.B.A.R.); 2 Structural and Synthetic Biology Research Centre, Malaysia Genome Institute, 43000 Bangi, Selangor, Malaysia

**Keywords:** enzymatic epoxidation, 1-nonene oxide, phenylacetic acid, lipase

## Abstract

Herein, an efficient epoxidation of 1-nonene is described. In a simple epoxidation system, commercially available Novozym 435, an immobilized *Candida antarctica* lipase B, and hydrogen peroxide (H_2_O_2_) were utilized to facilitate the *in situ* oxidation of phenylacetic acid to the corresponding peroxy acid which then reacted with 1-nonene to give 1-nonene oxide with high yield and selectivity. The aliphatic terminal alkene was epoxidised efficiently in chloroform to give an excellent yield (97%–99%) under the optimum reaction conditions, including temperature (35 °C), initial H_2_O_2_ concentration (30%), H_2_O_2_ amount (4.4 mmol), H_2_O_2_ addition rate (one step), acid amount (8.8 mmol), and stirring speed (250 rpm). Interestingly, the enzyme was stable under the single-step addition of H_2_O_2_ with a catalytic activity of 190.0 Ug^−1^. The entire epoxidation process was carried out within 12 h using a conventional water bath shaker.

## 1. Introduction

Epoxides are highly important intermediates for the production of a variety of useful commercial products [1]. Due to the ring strain and polarity of the oxirane group, epoxides are highly reactive for the synthesis of various desirable functional groups [2]. In this study, we highlight our immense interest in the chemistry and properties of 1-nonene oxide, which was recently been used as an important ingredient in formulating (a) a surfactant-like aminoalcohol lipoid to improve microparticle solubilization and transportation in pharmaceutical drug delivery [3], (b) bioactive compounds like achaetolide to exhibit the antibacterial and cytotoxic properties in asymmetrical synthesis [4], and (c) a lipopeptide-like hermitamide to displace and inhibit sodium channel activity in clinical applications [5,6].

Many oxidations of 1-nonene using metal catalysts or *meta*-chloroperoxybenzoic acid (*m*-CPBA) have been reported [5,7]; however, the hazardous nature of these methods and the tendency of epoxides to be hydrolyzed prevent their use on a large scale [8,9]. As a means of addressing these problems, the use of an environmentally benign biocatalyst like Novozym 435 together with the employment of H_2_O_2_ have become a viable and interesting idea on both economic and environmental grounds [10–12]. Over the years, researchers have sought many efficient enzymatic techniques in epoxide preparations, for example, the employment of dimethyl carbonate and ethyl ester as perhydrolysis substrates and as solvents [13]. Nevertheless, such systems have shown disadvantages in lipase stability due to the formation of water-soluble short-chain co-products (acetic acid and methanol) which inhibit enzyme activity [14].

To overcome such problems, the advent of new perhydrolysis substrates like lactone have led to improved enzyme activity and served as efficient oxidation systems for several alkenes [15]. However, the reaction time and H_2_O_2_ concentration that are required to epoxidise some alkenes like styrene are less practical in industrial applications. In addition to that, lipase was reported to be deactivated by the oxidation system containing H_2_O_2_ (50%) and ethyl acetate; therefore, special attention is needed to increase the activity and stability of the enzyme by developing a more efficient epoxidation protocol. Recently, we have reported some of our findings on chemoenzymatic epoxidation of 1-nonene [16]. A variety of inexpensive perhydrolysis substrates was screened for the reaction, and phenylacetic acid was found to give the best outcome. At the same time, the effect of solvents, enzyme sources, and enzyme concentrations was also reported. As part of our ongoing research, an improvement of the epoxidation system was carried out by utilizing phenylacetic acid as the perhydrolysis substrate, since no water-soluble acid or alcohol is generated during its perhydrolysis reaction. This paper also focused on the investigation of various reaction parameters for the chemoenzymatic epoxidation of 1-nonene, with the aim of determining the optimal reaction conditions with regard to enzyme stability and reaction efficiency, thus resulting in high yield and selective epoxides in a short reaction time.

## 2. Results and Discussion

**Scheme 1:**
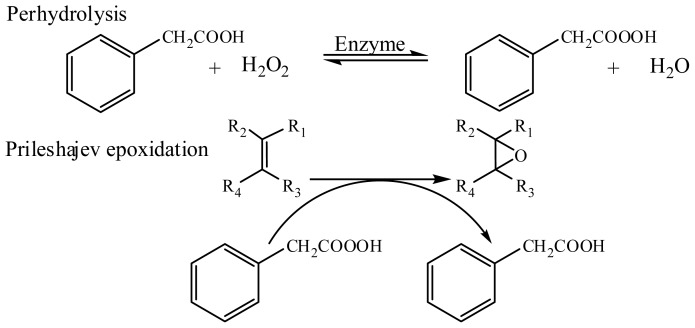
Illustration of the chemoenzymatic epoxidation of alkenes by lipase-catalyzed perhydrolysis of phenylacetic acid, which then epoxidises the double bonds in the alkene via the Prileshajev mechanism.

In a chemoenzymatic epoxidation reaction, the enzyme is the most important tool for the formation of peroxy acids from the perhydrolysis reaction between the carboxylic acid and oxidant, such as H_2_O_2_, which then donates an oxygen atom to the double bond in the alkene, giving the respective epoxide and regenerating the acid [10] (Scheme 1).

### 2.1. Influence of Temperature

**Figure 1:**
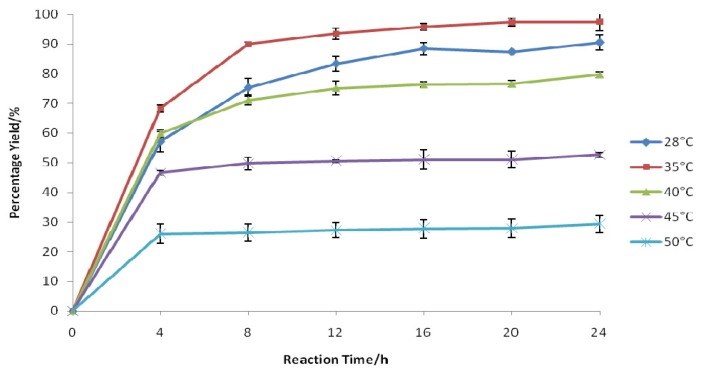
Effect of reaction temperatures on the synthesis of 1-nonene oxide in 10 mL of chloroform. A mixture of 1-nonene (0.6 mmol), phenylacetic acid (7 mmol), H_2_O_2_ (30% *w*/*w*, 3.1 mmol), and Novozym 435 (1.4% *w*/*w*, 16 mg) was shaken in a water bath shaker at 200 rpm for 24 h. The H_2_O_2_ solution was added to the reaction mixture dropwise every 15 min over a period of 1 h.

The study of the effect of temperature is essential as temperature changes can have a great influence on substrate solubility as well as have a direct effect on the reaction rate and enzyme catalytic properties [17]. Moreover, an increase in temperature reduces the mass transfer limitation, which results in an increased yield [18]. In chloroform, the highest yield (97%) was achieved when a temperature of 35 °C was used (Figure 1). In most studies, Novozym 435 has been shown to have high thermostability at temperatures between 40 °C and 60 °C [19,20]. However, in this study, a further increase in temperature, above 40 °C, led to a huge drop in the yield. A high temperature promotes the degradation of H_2_O_2_, such that peroxidation of acids does not take place [21]. For the following investigations, the operating temperature was fixed at 35 °C.

### 2.2. Effect of H_2_O_2_

According to Orellana-Coca (2005), an optimal combination of H_2_O_2_ and temperature is essential to complete the epoxidation process under solvent-free conditions [21]. This led us to explore the significance of initial H_2_O_2_ concentration, its different amount, and its rate of addition on the epoxidation of 1-nonene in more detail.

#### 2.2.1. Effect of Initial H_2_O_2_ Concentration

**Figure 2:**
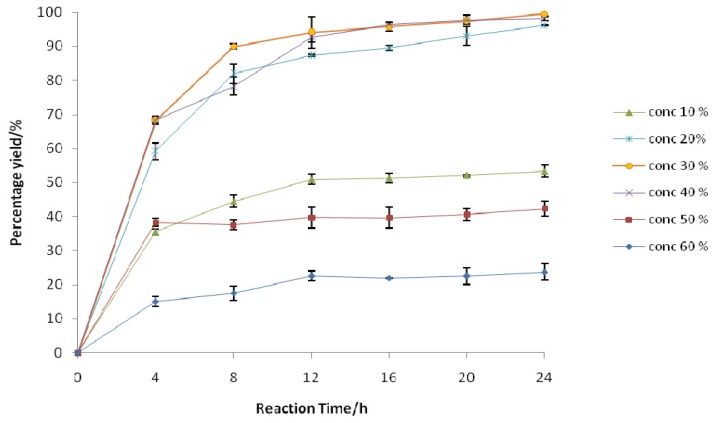
Graph illustrating the effect of H_2_O_2_ concentrations on the enzyme-catalyzed perhydrolysis reaction with 1-nonene (0.6 mmol), phenylacetic acid (7 mmol), and Novozym 435 (1.4% *w*/*w*, 16 mg) in 10 mL of chloroform at the optimal reaction temperature (35 °C) with shaking at 200 rpm for 24 h. The concentrations of H_2_O_2_ (% *w*/*w*, 3.1 mmol) were varied as specified in the graph. The H_2_O_2_ solution was added to the reaction mixture dropwise every 15 min over a period of 1 h.

A sufficient initial concentration of H_2_O_2_ with respect to the number of double bonds and the reaction conditions is necessary to facilitate the total conversion of alkenes within a short period of time [21]. The study was initiated by the preparation of a variety of H_2_O_2_ (*w*/*w*) concentrations (10%, 20%, 40%, and 50%) from commercially available sources (30% and 60%). A decrease in the H_2_O_2_ concentration (10%) led to slower epoxide formation (Figure 2). When a higher H_2_O_2_ concentration (50%–60%) was applied, the presence of 1-nonene oxide was observed in a very low yield, suggesting that the reaction was incomplete, perhaps due to enzyme inactivation [22]. A previous detailed investigation revealed that at H_2_O_2_ concentrations ranging from 6 to 12 M (or 18%–35%), the biocatalyst was only stable at 20 °C [23]. Moreover, in a recent study, Novozym 435 was deactivated at the H_2_O_2_ concentrations of 5 to 10 M (or 15%–30%) and temperature of 25 °C [24]. In comparison with our system, our results are in agreement with previous studies, whereby the combination of a temperature of 35 °C and an H_2_O_2_ concentration of 50%–60% resulted in a rapid loss of the lipase activity. In short, enzyme deactivation becomes more profound with a simultaneous increase in reaction temperature and H_2_O_2_ concentration.

#### 2.2.2. Effect of H_2_O_2_ Amount

**Figure 3:**
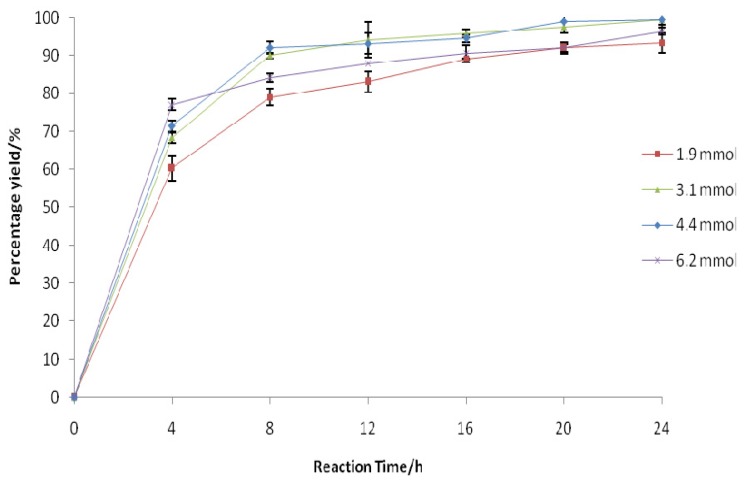
Effect of H_2_O_2_ amount on the enzyme-catalyzed epoxidation reaction. The reaction mixture comprised of 1-nonene (0.6 mmol), phenylacetic acid (7 mmol), H_2_O_2_ (30% *w*/*w*), and Novozym 435 (1.4% *w*/*w*) in 10 mL of chloroform under the optimum reaction temperature (35 °C), speed (200 rpm), and reaction time (24 h). The amounts of H_2_O_2_ were varied as follows: 1.9 mmol, 3.1 mmol, 4.4 mmol, and 6.2 mmol. The H_2_O_2_ solution was added to the reaction mixture dropwise every 15 min over a period of 1 h.

Although the highest yield of 1-nonene oxide could be achieved with a moderate H_2_O_2_ concentration (30%), a long epoxidation process of 24 h is economically valueless since it will affect the cost of production [25]. Moreover, at least a 10% stoichiometric excess of the required amount of H_2_O_2_ was used to compensate for its possible breakdown by light and temperature [21]. The influence of the amount of H_2_O_2_ on the epoxidation reaction was studied by varying the molar ratio of the alkene to H_2_O_2_ with the aim of obtaining a high epoxide yield within a short period of time. Within the first 4 h, the epoxidation rate increased as the amount of H_2_O_2_ increased from 1.9 mmol to 6.2 mmol (Figure 3). However, prolonging the reaction time to more than 4 h resulted in little further epoxide formation. The lipase may undergo a critical denaturation process due to its substantial interaction with excess H_2_O_2_ in the system [22]. Interestingly, the reaction performed with an amount of 4.4 mmol achieved the maximum yield (99%) within 20 h of reaction time. A further reduction in the reaction time is necessary to promote eco-friendly chemoenzymatic epoxidation on both the laboratory and industrial scales [11].

#### 2.2.3. Effect of the Rate of H_2_O_2_ Addition

The determination of an accurate dosing rate for H_2_O_2_ is one of the main steps in minimizing enzyme inactivation [10]. In this part of the study, we investigated potential ways to increase the turnover of epoxide in the shortest time; moreover, we tried to understand the requirements for the successive addition of H_2_O_2_ on this newly tailored epoxidation methodology. The experimental parameters were varied by adding the total amount of H_2_O_2_ in varying portions (one and four steps) over several periods of time (0 and 1 h) (data not shown). Surprisingly, a similar epoxide yield was obtained when H_2_O_2_ was added in one single addition compared to four additions. Thus, from a kinetic point of view, no clear advantage was found for stepwise addition [21] as the enzyme still retained its catalytic activity under the designed experimental conditions.

### 2.3. Effect of Phenylacetic Acid Amount

**Figure 4:**
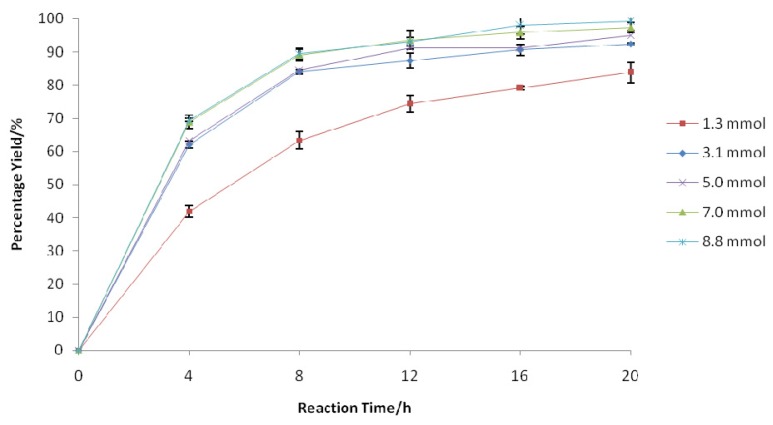
Effect of phenylacetic acid amount on the synthesis of 1-nonene oxide. The reaction mixture consisted of 1-nonene (0.6 mmol), H_2_O_2_ (30% *w*/*w*, 4.4 mmol), and Novozym 435 (1.4%, *w*/*w*) in 10 mL of chloroform at the optimum reaction temperature (35 °C) and speed (200 rpm) for 20 h. The amounts of phenylacetic acid were varied as follows: 1.3 mmol, 3.1 mmol, 5.0 mmol, 7.0 mmol, and 8.8 mmol. The H_2_O_2_ solution was provided in a single addition to the reaction mixture.

Increasing substrate accessibility to the enzyme catalytic site can actually enhance the reaction, thus affording a higher yield [18]. Since the lipase-catalyzed perhydrolysis reaction is reversible, one substrate is often used in excess in order to shift the equilibrium towards synthesis [26]. There was a clear increase in the initial reaction rate with an increasing phenylacetic acid amount from 1.3 mmol to 8.8 mmol, such that complete epoxidation was achieved within 16 h of reaction time (Figure 4). Furthermore, this experiment pointed out that increasing the amount of phenylacetic acid did not result in significant deactivation of Novozym 435 under the design experimental conditions. Therefore, for further optimization, an amount of 8.8 mmol was selected.

### 2.4. Effect of Stirring Rate

Stirring rate is an essential parameter, as this factor substantially enhances mass transfer to the active site of the enzyme and facilitates product release from the enzyme [26]. The results indicate that the highest stirring rate (250 rpm) for the water bath shaker provided the optimum conditions, which gave a maximum yield of 99% (data not shown). Since there was almost no notable difference in yield between the reaction times of 12 h and 16 h, 12 h was chosen as the best condition for the synthesis of 1-nonene oxide at 250 rpm. Although the influence of stirring rate in our designed experiment had a minor effect on the lipase-mediated synthesis of 1-nonene oxide, the overall reaction time was successfully reduced to 12 h by modifying the stirring rate (250 rpm), enabling a facile route to the chemoenzymatic industrial process.

## 3. Experimental Section

### 3.1. Materials

All the chemicals and solvents were of analytical reagent grade, unless specified otherwise. 1-Nonene (98%) was obtained from Sigma-Aldrich (St. Louis, MO, USA). Chloroform and H_2_O_2_ (60%, *w*/*w*) were purchased from Fisher Scientific (Leicester, UK). Dichloromethane, H_2_O_2_ (30%, *w*/*w*), and phenylacetic acid were purchased from Merck (Darmstadt, Germany). A commercially available enzyme, *Candida antarctica* lipase B immobilized on a macroporous acrylic resin (Novozym 435), was obtained from the Novozymes corporation (Bagsvaerd, Denmark). The standard for 1-nonene oxide was prepared and identified according to the literature [13]. For a GC-MS analysis, ethyl acetate (HPLC grade) was acquired from Fisher Scientific (Leicester, UK).

### 3.2. Methods

#### 3.2.1. General Synthetic Procedure for Chemoenzymatic Epoxidation

The epoxidation reaction was carried out in a 50-mL round-bottomed flask containing the alkene (0.6 mmol), organic solvent (10 mL), and specified amounts of phenylacetic acid, H_2_O_2_, and Novozym 435. The reaction was initiated by H_2_O_2_ which was added into the system using an autotitrator (Metrohm, New Zealand). All the experiments were carried out at least in duplicate using a water bath shaker from Hotech Instruments (Taiwan). Control experiments were prepared and treated in the same way; however, no background epoxidation reaction was observed. 0.1 mL of the sample was withdrawn intermittently from the reaction mixture and diluted in 9.9 mL of an organic solvent (ethyl acetate; HPLC grade) for subsequent analysis with GC-MS. The yield was determined based on the external standard calibration plot equation of the corresponding epoxide. The reaction mixture was treated with excess water (50 mL, twice) followed by washes with 1.0 M NaOH. The organic layer phase was separated from the aqueous layer and was then dried over 5% Na_2_SO_3_ and Na_2_SO_4_ for 1 h. The filtered solution was evaporated under vacuum distillation. The purified epoxide was then characterized by an FT-IR, GC-MS, and ^1^H and ^13^C NMR, and compared with known spectra.

#### 3.2.2. Analytical Procedure

The epoxide yields were determined using a GC from Agilent Technology (model 7890, Wilmington, DE, USA) equipped with an HP-5 ms column (30.0 m × 0.25 mm × 0.25 μm) and an MS detector (model 5975 C inert-MSD) with a triple axis. The machine was operated in electron ionization (EI) mode with an ionizing energy of 70 eV. An injection of 1 μL was made in splitless mode with the inlet temperature set at 280 °C. The MS detector was operated at a thermal aux temperature of 280 °C with helium as the carrier gas at a flow rate of 0.9 mL/min. The results were acquired separately by using scanning (SCAN) mode for qualitative analysis and selective ion monitoring (SIM) mode for quantitative analysis.

The GC oven temperature was set at an initial temperature of 50 °C for 0 min, then raised to 260 °C at 10 °C/min and held for 5 min. A full scan of the mass spectrum was performed in the *m/z* SCAN range of 45–150 amu. Retention times of 3.6 min for 1-nonene and 5.7 min for 1-nonene oxide were identified. For the SIM method, ions in 1-nonene and 1-nonene oxide were observed at fragmentations of *m*/*z* = 55.1, 56.1, 69.1, and 70.1 and *m*/*z* = 55.1, 56.1, 58.1, 69.1, and 71.1, respectively.

#### 3.2.3. Characterization

Characterization of the final epoxide product was performed using various spectroscopic analyses and compared with the published spectra. Infrared spectra were obtained using FT-IR (Perkin Elmer, model 1650, Norwalk, CT, USA) with a technique known as universal attenuated-total reflectance (UA-TR). The ^1^H and ^13^C NMR spectra were recorded at room temperature using NMR (JOEL JNM-ECA, Tokyo, Japan) spectrometer with CDCl_3_ as the solvent and tetramethylsilane (TMS) as the internal standard.

#### 3.2.4. Spectral Data

1-Nonene oxide [5]: Yield, 99%; Pale yellowish viscous oil; IR v max (cm^−1^): 2927, 2857, 1011, 835; ^1^H-NMR (400 MHz) δ 0.86–0.9 (t, *J* = 6.42 Hz, 3H, C*H*_3_), 1.1–1.6 (m, 12H, C*H*_2_-C*H*_2_), 2.45–2.49 (dd, *J* = 2.75, 5.58 Hz, 1H, CHOC*H*_2_), 2.74–2.77 (dd, *J* = 3.67, 5.48 Hz, 1H, CHOC*H*_2_), 2.88–2.94 (m, 1H, C*H*OCH_2_); ^13^C-NMR (100 MHz) δ 14.0, 22.6, 25.9, 29.2, 29.4, 31.7, 32.5, 47.1, 52.4; MS *m*/*z* (rel. int.): 71 (100), 55.1 (61), 56 (40), 69 (40), 58 (37), 68 (33), 81 (32), 67 (30), 57 (29), 85 (13), 99 (10), 113 (5), 142 [M^+^] (0.1).

## 4. Conclusions

The developed system exhibited several advantages, such as high yield, mild operating temperature, low by-product formation, and high enzyme stability, over other available methods in the literature. This study demonstrated that chemoenzymatic epoxidation is feasible with a single addition of H_2_O_2_, providing operational lifetime/stability of lipase in industrial applications. It also demonstrates the advantages of this lipase-mediated oxidation technique to obtain a variety of highly useful products. Increasing the phenylacetic acid supply for the reaction resulted in increased epoxide formation, showing that a different peroxy acid is generated for the lipase-catalyzed epoxidation system, as no strong acid, which is detrimental to the enzyme, is generated during the perhydrolysis process. A considerable reduction in the reaction time was achieved through this newly designed chemoenzymatic epoxidation reaction, providing a practical epoxidation system with a high yield in a sustainable manner. In addition, studies on the enantioselectivity of the epoxide and efforts involving recycling the used phenylacetic acid are currently ongoing in our laboratory.
